# Supplementary figures and images for: HMP-S7 Is a Novel Anti-Leukemic Peptide Discovered from Human Milk
Source: Biomedicines. 2021 Aug 9;9(8):981. doi: 10.3390/biomedicines9080981 (PMC8394283; doi:10.3390/biomedicines9080981)

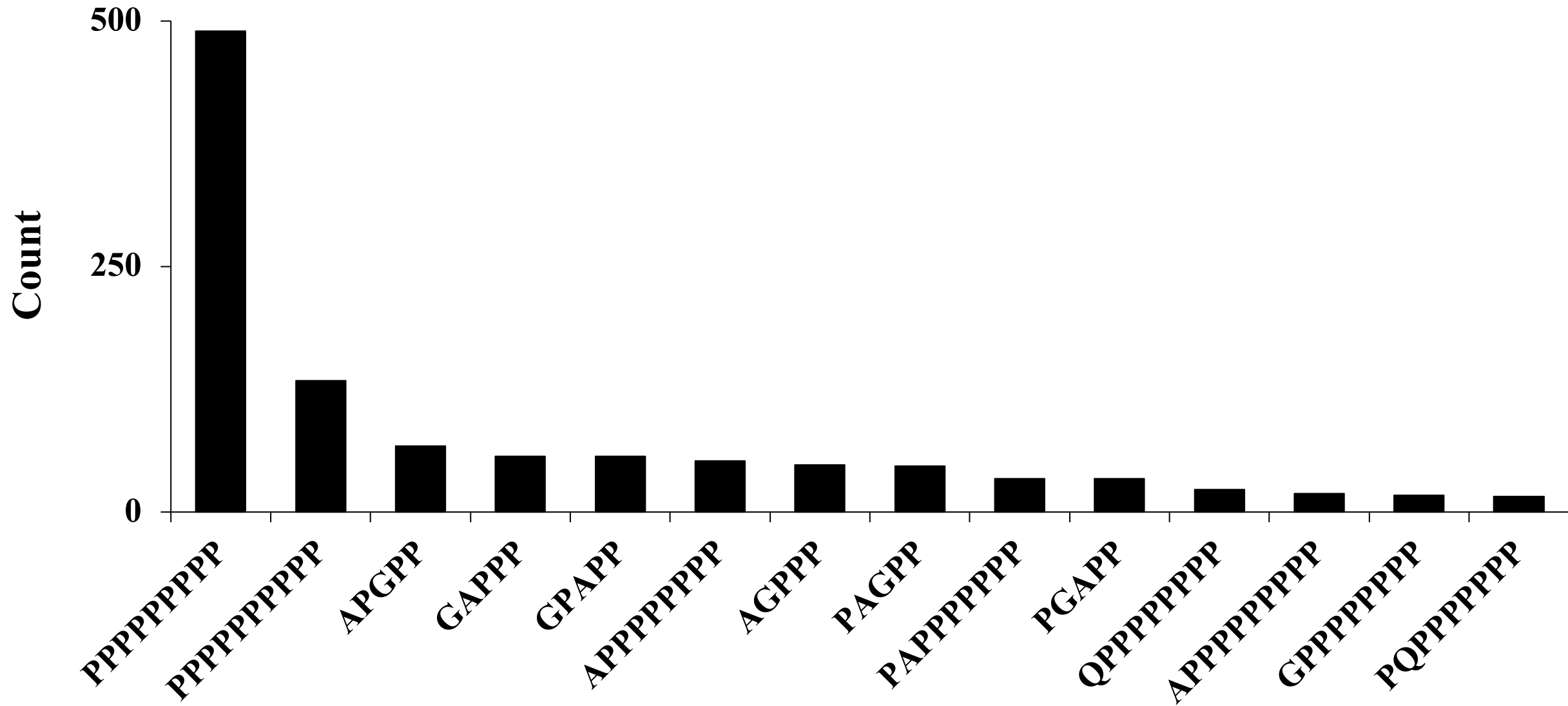

**Figure S1:** The frequency of the identified peptides with proline-rich sequences.

Supplement: Supplementary file 1 [file biomedicines-09-00981-s001.zip › biomedicines-1308314-supplementary/Supplementary information/Fig S1_R1.pdf]
